# Differences between occupational and non-occupational-related motor vehicle collisions in West Virginia: A cross-sectional and spatial analysis

**DOI:** 10.1371/journal.pone.0227388

**Published:** 2019-12-31

**Authors:** Toni Marie Rudisill, Sreyas Menon, Brian Hendricks, Motao Zhu, Gordon S. Smith

**Affiliations:** 1 Department of Epidemiology, West Virginia University, Morgantown, West Virginia, United States of America; 2 School of Medicine, West Virginia University, Morgantown, West Virginia, United States of America; 3 Center for Injury Research and Policy, Abigail Wexner Research Institute at Nationwide Children’s Hospital, Columbus, Ohio, United States of America; Tsinghua University, CHINA

## Abstract

**Background:**

Motor vehicle collisions comprise the majority of occupational-related fatalities in the United States and West Virginia has one of the highest occupational-related fatality rates in the nation. The purpose of this study was to compare work and non-work-related collisions, crash locations, and the characteristics of in-state and out-of-state drivers ≥18 years of age who were fatally injured in work-related collisions in West Virginia.

**Methodology:**

Data were from the 2000–2017 Fatality Analysis Reporting System. Work and non-work-related crashes and characteristics in-state vs. out-of-state drivers were compared using binary and multivariable logistic regression analyses. Crash locations were compared via spatial analyses using kernel density estimations.

**Results:**

Among the 5,835 individuals fatally injured in crashes, 209 were designated ‘at work’. The odds of being a work-related crash were 85% lower [Odds Ratio (OR) = 0.15; 95% confidence interval (CI): 0.04, 0.49] among those testing positive for alcohol, but 2.5 times greater (OR = 2.56; 95% CI: 1.16, 5.65) among those holding a commercial driver’s license. The odds of being an in-state driver were 75% lower (OR = 0.25; 95% CI: 0.12, 0.53) among those wearing a safety belt, but 2.7 times greater among workers testing drug positive (OR = 2.67; 95% CI: 1.10, 6.52). In-state drivers were also less likely to be driving a large truck or be involved in single vehicle collisions and less likely to experience crashes on weekends, nights, or on highways. Spatial patterns of crash locations varied slightly between workers and non-workers.

**Conclusions:**

Work-related crashes differed greatly from non-work-related crashes in West Virginia. Stark differences existed between in-state and out-of-state workers and their crashes. Various avenues for workplace safety interventions exist, including seatbelt initiatives and drug testing policies for non-commercial drivers, which could help mitigate West Virginia’s elevated, occupational-related, traffic fatality rate.

## Introduction

According to the Bureau of Labor Statistics, there were 5,147 work-related fatalities in the United States (U.S.) in 2017 alone [1]. Previous studies have shown that certain areas of the country experience higher rates of work-related injuries and fatalities compared to others, with the southeastern U.S. being the most affected [2]. West Virginia (WV), a southeastern Appalachian state, has one of the highest fatal occupational injury rates in the country. In 2017, WV’s fatal occupational injury rate was the fourth highest among states at twice the national average (e.g. 7.4 per 100,000 vs. 3.5 per 100,000 full-time equivalent workers nationally) [1]. WV’s economy is heavily dependent on the mining, logging, and transportation industries, which also happen to be some of the most dangerous occupational sectors [1, 3, 4].

While work-related injuries and fatalities are caused by a myriad of reasons, transportation-related incidents account for a vast majority (e.g. 40%) of occupational-related deaths nationally [4]. Previous studies have found that law enforcement, construction workers, mining, natural gas/oil extraction workers, and truck drivers are more likely to die in motor vehicle collisions while on the job [5–11]. Previous studies specifically of WV workers have also found that transportation incidents were the leading cause of work-related death [12]. Numerous studies and systematic reviews have elucidated risk and protective factors associated with occupational-related motor vehicle collisions including extended work shifts, vehicle defects, vehicle type or configuration, drivers’ health, driver fatigue, drivers’ drug and alcohol use, drivers’ age and sex, the amount the employee drives, occupational stress, the type of road the employee drives, and the time of day [10, 11, 13–21]. Other studies of commercial truck drivers have found that many individuals speed and violate Hours of Service regulations in order to meet perceived unrealistic delivery schedules [15].

Despite the known risk factors associated with occupational injuries/fatalities resulting from motor vehicle collisions, driving in Appalachia, especially for work, may be inherently dangerous. West Virginia is the only state to lie completely within the Appalachian region. Previous studies have shown that the traffic fatality rate in Appalachia and WV is 40% and 70% higher, respectively, than the national rate [22, 23]. The fatality rate in WV for large trucks, which are typically driven only for work, is nearly 1.75 times higher than the national average [23]. While the exact reason for this disparity in WV is unknown, several studies have proposed potential explanations. Seat belt usage in Appalachia is known to be lower than the rest of the country [24]. Previous studies have also suggested that drivers’ speeding, lower seatbelt use, and drivers’ increased drug and alcohol use may contribute to the elevated traffic fatality rates [25]. Another study has suggested that drivers’ opioid use, which is particularly elevated in the WV population, as a potential risk factor for collision [26]. Other factors such as the state’s curvy topography, rurality, geographic isolation, and distance to emergency medical care may compound WV’s high fatality rates [23, 24].

Because motor vehicle collisions are the biggest contributor of occupational-related fatalities both nationally and in WV, the purpose of this study was to assess the characteristics of work-related crashes compared to non-work-related collisions in WV. The objective was to discern what factors predict work-related crashes and to determine where they most often occur within the state. Additional analyses were conducted to compare driver, vehicle, and crash characteristics of in-state vs. out-of-state drivers who were killed in work-related collisions. It was hypothesized that out-of-state drivers may be fundamentally different from in-state drivers as they may be unfamiliar with WV’s rurality, geography, and road systems. Previous studies have shown that rural collisions and individuals holding out-of-state drivers’ licenses are more likely to be killed in crashes [17, 27]. These findings could potentially inform public safety initiatives or employer safety programs.

## Materials and methods

### Subjects

Data were obtained from the Fatality Analysis Reporting System (FARS). FARS is a publicly available national database maintained by the National Highway Traffic Safety Administration (NHTSA). FARS is a compilation of all fatal crashes which occur on U.S. public roadways where any individual whom is involved in the crash dies within 30 days of the incident. All states and U.S. territories contribute to FARS. FARS contains well over 100 variables pertaining to circumstances surrounding the crash, vehicles, and any individual involved. FARS has been described previously in detail elsewhere [28, 29].

The study population was limited to any individual (i.e. driver, pedestrian, or passenger) ≥ 18 years of age who was fatally injured in a motor vehicle collision within WV’s state boundary from Jan 1, 2000 to December 31, 2017. The purpose of limiting the age range was because no individual who was considered ‘at work’ at time of collision was <18 years of age. This study was approved by West Virginia University’s Institutional Review Board.

### Data management

Numerous independent variables were created using the FARS data (Table 1). These specific variables were chosen based on findings from previous transportation and/or occupational research [10, 11, 13–21]. With regards to race, the “Other” category included any individual deemed non-Caucasian by first responders. An individual was considered to have a commercial drivers’ license (CDL) regardless if it was current, suspended, or revoked at time of collision. Individuals were considered to be wearing a safety belt regardless if it was a shoulder and lap harness, lap only, or shoulder only at time of collision. The body type of the vehicle involved in the collision was categorized as a large truck, light truck or van, or passenger car based on NHTSA’s recommended categorization [28]. Vehicle age was determined by subtracting the year of the crash from the model year of the vehicle the person was driving at time of crash. Air bags were considered to be deployed (yes/no) regardless if it was the front and/or side air bags that deployed during the crash. Individuals were considered ‘ejected’ whether they were fully or partially ejected from the vehicle during the collision. An individual was considered to be positive for alcohol if their blood alcohol concentration was ≥0.01 g/ml. An individual was considered drug positive if their toxicology tests detected any substance. Individual drugs and drug categories were also reviewed. An individual was categorized as having been cited (yes/no) for a traffic violation within the past 5 years. This included previous collisions, license suspensions, driving while intoxicated convictions, speeding tickets, or any other type of traffic violation. Season when the collision occurred was based on the meteorological classification. Weekend collisions (yes/no) were those which occurred either on Saturdays or Sundays. Nighttime collisions were classified as those occurring anytime between 5:00 PM and 4:59 AM (yes/no). Weather conditions were considered inclement if any precipitation or excessive wind was occurring at time of the collision. Collisions were classified as to whether they occurred on a state or federally designated highway (yes/no). If there was evidence that the vehicle rolled over during the collision, this was binary coded (yes/no). If the collision occurred on a road with a speed limit ≥55 miles per hour, this was considered a high-speed crash (yes/no). The roadway in which the crash occurred was also coded if it was straight or curved or level or sloped.

**Table 1:** Characteristics of individuals ≥18 years of age who were fatally injured in motor vehicle collisions by work status in West Virginia, 2000–2017 (N = 5,835)^a^.

	Work-related		Non-work-related		Total	
Characteristic	N	%	N	%	N	%
Person type						
Driver	177	84.7	4,180	74.3	4,357	74.7
Passenger	16	7.6	1,094	19.5	1,109	19.0
Pedestrian	16	7.7	352	6.3	369	6.3
Male						
Yes	198	94.7	3,970	70.6	4,168	71.4
No	11	5.3	1,656	29.4	1,667	28.6
Age group (years)						
18–34	42	20.1	2,171	38.6	2,213	37.9
35–54	109	52.2	1,796	31.9	1,905	32.7
≥55	58	27.8	1,659	29.5	1,717	29.4
Race						
White	190	91.4	5,455	97.2	5,645	97.0
Other	18	8.7	158	2.8	176	3.0
Missing	1		13		14	
Commercial drivers’ license						
Yes	131	70.0	568	10.8	699	12.9
No	56	30.0	4,684	89.2	4,740	87.1
Missing	22		374		396	
Safety belt in use						
Yes	66	44.9	1,471	35.6	1,537	35.9
No	81	55.1	2,663	64.4	2,744	64.1
Missing	62		1,492		1,554	
Driver’s license state						
West Virginia	93	48.4	4,311	81.9	4,404	80.7
Outside of West Virginia	99	51.6	956	18.1	1,055	19.3
Missing	17		359		376	
Vehicle type						
Large truck	130	62.2	10	0.2	140	2.4
Light truck or van	49	23.4	1,982	35.3	2,031	34.8
Passenger car	11	5.3	2,462	43.8	2,473	42.4
Other	19	9.1	1,166	20.8	1,185	20.3
Missing	0	0	6		6	
Vehicle ≥ 10 years of age						
Yes	47	22.5	2,135	37.9	2,182	37.4
No	162	77.5	3,491	62.1	3,653	62.6
Airbag deployed						
Deployed	15	9.3	1,054	22.2	1,069	21.8
Not deployed	146	90.7	3,685	77.8	3,831	78.2
Missing	48		887		935	
Victim ejected during crash						
Ejected	52	27.2	1,439	29.4	1,491	29.3
Not ejected	139	72.8	3,459	70.6	3,598	70.7
Missing	18		728		746	
Alcohol detected						
Yes	8	4.2	1,820	36.2	1,828	35.1
No	184	95.8	3,204	63.8	3,388	64.9
Missing	17		602		619	
Drug(s) detected						
Yes	28	14.7	1,565	31.7	1,593	31.1
No	162	85.3	3,368	68.3	3,530	68.9
Missing	19		693		712	
Traffic citation < 5 years						
Yes	76	40.0	1,979	38.0	2,055	38.1
No	114	60.0	3,229	62.0	3,343	61.9
Missing	19		418		437	
Survival time after crash (minutes)						
<2	63	30.9	874	16.1	937	16.6
2–120	118	57.8	3,214	59.2	3,332	59.1
≥120	23	11.3	1,346	24.8	1,369	24.3
Missing	5		192		197	
Single vehicle crash						
Yes	141	67.5	3,381	60.1	3,522	60.4
No	68	32.5	2,245	39.9	2,313	39.6
Season (meteorological)						
Winter	51	24.4	1,168	20.8	1,219	20.9
Spring	40	19.1	1,355	24.1	1,395	23.9
Summer	64	30.6	1,618	28.8	1,682	28.8
Fall	54	25.8	1,485	26.4	1,539	26.4
Weekend						
Yes	23	12.0	1,750	33.2	1,773	32.4
No	169	88.0	3,524	66.8	3,693	67.6
Missing	17		352		369	
Night						
Yes	75	36.6	2,979	54.2	3,054	53.6
No	130	63.4	2,516	45.8	2,646	46.4
Missing	4		131		135	
Inclement weather						
Yes	44	22.9	899	17.1	943	17.3
No	148	77.1	4,370	82.9	4,518	82.7
Missing	17		357		374	
Highway collision						
Yes	175	83.7	3,766	67.0	3,941	67.6
No	34	16.3	1,859	33.0	1,893	32.4
Missing	0		1		1	
Number of lanes						
1	1	0.5	45	0.9	46	0.8
2	177	92.2	4,948	93.9	5,125	93.9
≥3	14	7.3	276	5.2	290	5.3
Missing	17		357		374	
Vehicle rollover						
Yes	108	56.0	1,702	32.3	1,810	33.1
No	85	44.0	3,566	67.7	3,651	66.9
Missing	16		358		374	
High speed limit road (≥55 miles per hour)						
Yes	162	85.3	3,127	61.4	3,289	62.2
No	28	14.7	1,970	38.7	1,998	37.8
Missing	19		529		548	
Road alignment						
Straight	87	45.3	2,841	54.0	2,928	53.7
Curve	105	54.7	2,424	46.0	2,529	46.3
Missing	17		361		378	
Road slope						
Level	87	45.3	3,066	58.3	3,153	57.8
Grade	99	51.6	1,935	36.8	2,034	37.2
Hillcrest	4	2.1	215	4.1	219	4.1
Sag (Bottom of hill)	2	1.0	46	0.9	48	0.9
Missing	17		364		381	

^a^: Percentages may not add to 100% due to rounding.

### Statistical analyses

Separate multivariable logistic regression analyses were conducted to assess odds of fatal occupational and non-occupational related collision among drivers, and subsequent in-state vs. out-of-state fatal occupational related collisions among drivers. In order to determine what factors predicted these outcomes, a model building process was undertaken to identify the most parsimonious model. Binary relationships between each of the separate model outcomes and independent variables were assessed [30]. Any independent variable that was significant with work status at a p-value ≤ 0.20 level in the binary models was considered for a multivariate logistic regression model.[31] Variables were kept or removed from the multivariate logistic model based on backward selection technique with the p-value ≤0.15; this p-value was chosen as it has been shown to be the most effective among models using backward selection technique to ensure variables which are important to the model or are potential confounding variables remain within the model [32]. Akaike Information Criterion and Hosmer Lemeshow Goodness-of-Fit test were assessed to evaluate model fit. All statistical analyses were conducted using SAS software version 9.4 (Cary, North Carolina).

For spatial analysis, geographic patterns of work and non-work-related crashes were analyzed separately using dot density maps and isotropic kernel density estimation (KDE) using each event’s latitude and longitude and a WGS84 projection in the spatstat R package [33]. Briefly, KDE is a non-parametric statistical approach for extrapolating point data over larger geographic areas, and useful in limiting bias due to Modifiable Areal Unit Problems [34]. Point density functions, or intensity, was calculated using a fixed bandwidth with a Gaussian kernel, and included the Jones-Diggle improved edge correction shown to reduce distortion of density estimates nearer to the boundary, which in this case was the WV state boundaries [35, 36]. Adaptive and fixed bandwidths for KDE analyses have been explored in previous public health research [34, 37, 38]. In general, fixed bandwidths are ideal where estimating density an event, but are potentially insufficient for risk estimation particularly in sparsely populated areas [34, 39, 40]. For this study, the primary concern was geographic distance and number of events (e.g. not risk), therefore fixed bandwidth appropriately represents density of work and non-work-related crashes [34].

## Results

There were 5,835 individuals fatally injured in motor vehicle collisions which occurred on WV roads from 2000–2017 (Table 1). Among these individuals, 209 (3.6%) were fatally injured while ‘at work’ and the majority of individuals were drivers (84.7%) of the vehicles involved. Most fatally injured workers were white (91.4%), males (94.7%), and aged 35–54 years (52.2%) which contrasted slightly from non-workers whom tended to be younger (38.6%). The majority of workers held a CDL (70%) and 48% were WV residents. While most of those involved in non-work-related collisions were occupants of either passenger cars (43.8%) or light trucks/vans (35.3%), most workers were occupants of large trucks, which includes tractor trailers (62.2%). While safety belt usage rates were low for both workers and non-workers, workers tended to use safety belts slightly more (44.9% for workers vs. 35.6% for non-workers). Compared to non-workers, those killed in work-related crashes tended to test positive less for drugs (14.7%) and alcohol (4.2%). Those ‘at work’ tended to expire quicker than non-workers with nearly 31% dying on impact compared to 16% for non-workers. However, vehicles carrying workers tended to be newer as only 22.5% of these vehicles were ≥10 years of age. In general, fatal crashes occurred more frequently during the week (e.g. Monday-Friday). While work-related crashes occurred mostly during the day (63.4%), non-work-related crashes were mostly at nighttime (54.2%). Weather was a factor in 23% of work-related crashes as opposed to 17% non-work collisions. Regardless of work status, most of the fatal crashes occurred on highways. However, highway collisions were more frequent in work-related crashes (84%) compared to non-work-related crashes (67.0%). A curved road alignment was the slightly more common setting of work-related crashes (54.7%), while a straight road alignment was the more common for non-work-related crashes (54.0%). While work-related crashes occurred with greater frequency on a grade (51.6%) than other road slopes, a grade was less common for non-work-related crash fatalities (36.8%).

### Work-related vs. non-work-related crashes

Differences in the odds of fatal collision for drivers involved in work vs. non work-related collisions are highlighted in Table 2. The odds of being a work-related fatal collision was predicted by drivers’ sex, alcohol and drug test status, commercial driver’s license status, type of vehicle driven, number of vehicles involved, weather at time of crash, and vehicle age, which explained 59% of the variance (e.g. Max rescaled R square). The Akaike Information Criteria improved (e.g. decreased indicating better fit) from the initial to final model (i.e. from 410.4 to 392.3, respectively) and the Hosmer Lemeshow Goodness-of-Fit test indicated acceptable fit (p = 0.2171) of the final model. However, only alcohol test status, having a commercial driver’s license, vehicle type, weather, and vehicle age remained statistically significant (i.e. p ≤0.05) in the final model. The odds of being a work-related fatality were 85% lower for those testing positive for alcohol (0.15; 95% CI: 0.04, 0.49) compared to those who did not. Additionally, the odds of being a work-related fatality were 2.5 greater for individuals holding a CDL (OR = 2.56; 95% CI: 1.16, 5.65) and 2.5 times greater (OR = 2.51; 95% CI: 1.30, 4.83) for those driving in inclement weather. However, the odds of being a work-related fatality were 65% lower for individuals driving an older vehicle as well (OR = 0.35; 95% CI: 0.16, 0.77).

**Table 2:** Factors that predict fatal work-related vs. non-work-related motor vehicle collisions among drivers in West Virginia, 2000–2017.

Factor	Odds Ratio	95% Confidence Interval
Sex		
Male	2.25	0.92, 5.52
Female	1.00	Referent
Alcohol positive		
Yes	0.15	0.04, 0.49
No	1.00	Referent
Drug positive		
Yes	0.56	0.25, 1.19
No	1.00	Referent
Commercial drivers’ license		
Yes	2.56	1.16, 5.65
No	1.00	Referent
Vehicle type		
Passenger car	0.001	0.001, 0.003
Light truck/van	0.005	0.001, 0.02
Large truck	1.00	Referent
Other	0.003	0.001, 0.02
Single-vehicle collision		
Yes	0.61	0.32, 1.16
No	1.00	Referent
Inclement weather		
Yes	2.51	1.30, 4.83
No	1.00	Referent
Vehicle age > 10 years		
Yes	0.35	0.16, 0.77
No	1.00	Referent

### In-state vs. out-of-state work-related crashes

Factors associated with in-state work-related collisions are displayed in Table 3. The odds of being an in-state driver were 75% lower (OR = 0.25; 95% CI: 0.12, 0.53) for those wearing a safety belt. Additionally, the odds of being an in-state driver were 2.7 times greater (OR = 2.67; 95% CI: 1.10, 6.52) among those testing drug positive. Among in-state drivers, narcotics were the most common drug identified (N = 12; 63%). The odds of being an in-state driver were 82% (OR = 0.18; 95% CI: 0.09, 0.37) and 53% lower (OR = 0.47; 95% CI: 0.25, 0.88) among those driving a large truck or being involved in a single vehicle collision, respectively. In-state drivers were less likely to be fatally injured on weekends, at night, or on the highway.

**Table 3:** Factors associated with in-state drivers whom were fatally injured in work-related motor vehicle collisions in West Virginia, 2000–2017^a^.

Factor	Odds Ratio	95% Confidence Interval
Sex		
Male	0.37	0.07, 1.98
Female	1.00	Referent
Race		
White	16.05	2.06, 124.9
Other	1.00	Referent
Age		
18–34	1.54	0.69, 3.46
35–54	1.00	Referent
≥55	1.92	0.97, 3.80
Restraint use		
Yes	0.25	0.12, 0.53
No	1.00	Referent
Alcohol positive		
Yes	3.00	0.59, 15.32
No	1.00	Referent
Drug positive		
Yes	2.67	1.10, 6.52
No	1.00	Referent
Commercial drivers’ license		
Yes	0.26	0.13, 0.53
No	1.00	Referent
Large truck		
Yes	0.18	0.09, 0.37
No	1.00	Referent
Single-vehicle collision		
Yes	0.47	0.25, 0.88
No	1.00	Referent
Weekend		
Yes	0.34	0.13, 0.92
No	1.00	Referent
Inclement weather		
Yes	0.86	0.42, 1.75
No	1.00	Referent
Nighttime collision		
Yes	0.50	0.27, 0.93
No	1.00	Referent
Citation history		
Yes	0.73	0.40, 1.34
No	1.00	Referent
Highway collision		
Yes	0.22	0.06, 0.79
No	1.00	Referent
Roll-over collision		
Yes	0.86	0.47, 1.56
No	1.00	Referent
Road curvature		
Curved	0.59	0.32, 1.08
Straight	1.00	Referent
Vehicle age > 10 years		
Yes	2.44	1.18, 5.06
No	1.00	Referent

^a^: N = 177; in-state drivers = 87; out-of-state drivers = 90

### Spatial analysis of work-related vs. non-work-related collisions

**Fig 1:**
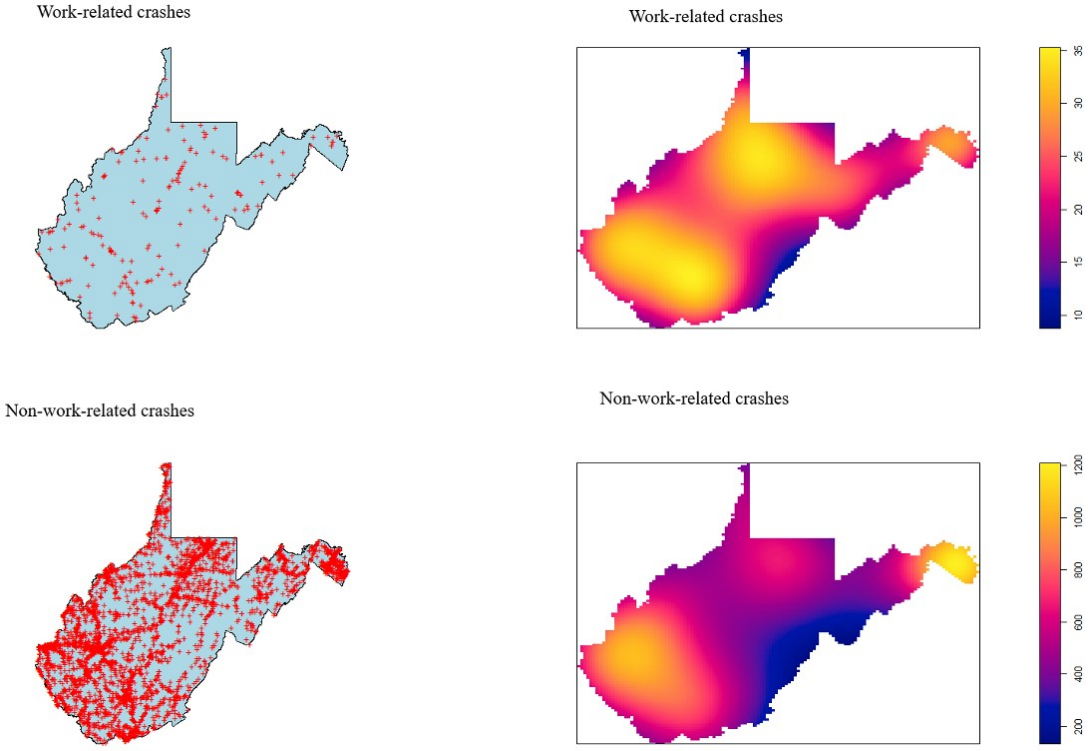
Location and kernel density estimation of work-related and non-work-related fatal collisions in West Virginia, 2000–2017. Upper-left: Location of work-related crashes; Upper-right: Kernel density estimation of work-related crashes. Lower-left: Location of non-work-related crashes; Lower-right: Kernel density estimation of non-work-related collisions. Areas of purple have low number of fatal crash sites, and bright yellow have a higher number of crash sites.

Dot density (left panels) and Kernel Density Estimation maps (right panels) are shown in Fig 1. Overall, there were more non-work-related crashes than work-related shown by the frequency of red “+” in the dot maps. For both types of crashes, density of dots clustered in south-central and in the northeastern regions of West Virginia. Despite generalities in point densities between crash types, work-related crashes had a relatively higher occurrence in southern and north central WV, indicated by bright yellow spots, as opposed to a single area of emphasis in the northeastern panhandle observed for non-work-related crashes.

## Discussion

The principle findings of this analysis were that fundamental differences existed not only between work-related and non-work-related fatal collisions which occurred in WV, but also among the in-state and out-of-state workers who were killed in these crashes. In general, the work-related collisions involved mostly middle-aged, non-impaired drivers of large trucks who were involved in single-vehicle collisions, on highways, during the daytime on weekdays. Non-work-related fatalities mostly involved younger occupants of passenger cars, light trucks, and vans. This analysis also revealed the existence of two major populations of workers killed on WV roadways. One population consisted of long-haul truck drivers, who were mostly non-WV residents. The other consisted of workers involved in local transport who were mostly residents of the state. While non-resident truck drivers made up a larger proportion of the work-related fatalities, workers who were residents of WV were found to have important risk factors for being fatally injured on the road. Resident workers were more likely to not wear a seatbelt, and were more likely to test positive for drugs, which has important workplace safety implications.

The findings of this analysis concerning work-related collisions were somewhat expected and supported by the extant literature. Previous U.S. studies have also determined that most work-related transportation fatalities involve white, male drivers between 25–55 years of age, driving large trucks on highways during daytime hours [10]. Previous national and international studies have also found that drivers killed on the job are generally not impaired by alcohol [13, 41]. Mandatory alcohol testing for large truck drivers by the Federal Motor Carrier Safety Administration (FMCSA) has resulted in a significant decline in the rate of alcohol involvement in fatal, U.S., large truck crashes [42]. It was also expected that workers were more likely to experience their collisions during inclement weather. In a 2010 survey conducted by the National Institute of Occupational Safety and Health of long-haul truck drivers, 24% of respondents said that they continued to drive despite fatigue, bad weather, or heavy traffic in order to meet timetables for the transport of loads. While traffic tends to decrease during adverse weather events, workers probably tended to stay on the roads to meet work demands while those driving for other purposes avoided this situation [43].

However, some findings of this analysis, especially those concerning the prevalence of single vehicle collisions and workers’ drug use, were slightly different than expected, but also explainable. Previous national studies of fatal work-related crashes have shown that multi-vehicle incidents are more prevalent than single vehicle collisions [10]. Previous literature has also found that workers driving large trucks are more likely to be killed in single-vehicle crashes, while occupants of smaller work vehicles are more likely to be killed in multiple vehicle collisions [11]. The reason why there were more single-vehicle collisions in WV could be due to having a higher proportion of large truck crashes in addition to the state’s topography and road systems. WV is mountainous and many roads—including highways—are narrow, steep, and curved. A large proportion of the work-related crashes occurred on grades and curved roads. While the cause of these collisions were often unknown, these drivers, who may be unfamiliar with WV’s roads, could easily have missed turns or ran off roads, which are common occurrences in single-vehicle collisions involving trucks [44].

As for drivers’ drug use, the high prevalence of stimulant use among truck drivers has been extensively studied in literature [45]. However, drug use was generally low among workers killed in WV and stimulant use was uncommon. Only in-state workers tended to test positive for drugs, but primarily for opioids. Drug use among large truck drivers may be lower due to tighter safety regulations, the institution of mandatory random drug screens, and hours of service restrictions. The prevalence of narcotics among in-state workers is likely due to the states’ high opioid use [26]. Opioids are one category of drugs known to negatively impact driving ability and crash rates [46].

While studies concerning in-state and out-of-state drivers are sparse in the extant literature, this analysis found that there are likely two distinct types of workers on WV roads. In contrast to out-of-state workers whom mainly drove large trucks, in-state workers tended to drive older, smaller vehicles, not hold a commercial driver’s license, and be involved in multiple vehicle collisions, on non-highways, during the daytime on weekdays. Thus, these results suggest that in-state drivers are likely driving more regionally and out-of-state drivers are likely carrying larger loads and potentially travelling greater distances. From a workplace safety perspective, these findings have numerous implications. For example, seatbelt usage was low for all fatally injured workers regardless of residence status, but more so for in-state residents; workplace safety policies or education of workers may be greatly needed as seatbelts are known to save lives. Additionally, employers of long-haul drivers may need to advise newer drivers/employees who travel on WVs roads and may be unfamiliar of the risk of collision considering the amount of crashes occurring in the northern and southern regions of the state. Protocols regarding inclement weather may also be needed as many large truck drivers met their demise during these episodes. Employers should consider adopting standard protocols for these situations and permit changes to operations and give drivers the ability to make decisions regarding the safety of the driving conditions without financial penalty. Moreover, this analysis revealed that the focus of work-related motor vehicle crash prevention efforts in WV should not focus solely on those who drive large trucks. The trucking industry is heavily regulated by the FMCSA and significant improvements to vehicle safety, drug and alcohol testing practices, and limitations on driving hours have been made. These regulations, however, do not apply to drivers of smaller vehicles that do not require a CDL, which was the case for many in-state workers. Also, due to the amount of drug positive resident workers, companies employing drivers of smaller fleet vehicles may want to consider random or mandatory drug testing programs. Currently, these practices exist for large trucking operations. In 2018, the FMCSA began to test for certain semi-synthetic opioid (e.g. hydrocodone, hydromorphone, oxycodone, and oxymorphone) in urine drug screens [47]. The detection of any scheduled controlled substance without a prescription disqualifies a driver operating a commercial motor vehicle. However, a letter from the prescribing physician assuring that the driver is safe to operate a commercial motor vehicle while a driver is taking an impairing medication as directed is sufficient to waive this disqualification [47]. Thus, even physicians who care for patients who drive smaller, non-commercial vehicles for work may want to consider advising their patients on the safety of certain medications known to affect driving ability.

### Limitations

While this was the first study to investigate in-state and out-of-state drivers involved in fatal work-related collisions in WV and crash location spatially, this analysis is not without limitation. For example, there are well-known limitations of drug and alcohol test results in FARS which have been described in detail [48]; however, WV is one of the best states for conducting and reporting drug and alcohol test results on individuals and has one centralized Medical Examiner’s Office [25]. Secondly, the FARS data do not provide details regarding the occupation/industry of those fatally injured. This information would illuminate more details regarding occupational risk factors for road fatalities in WV. While these data are available in the Census of Fatal Occupational Injuries (CFOI) database maintained by the U.S. Bureau of Labor Statistics, only limited statistics are publicly available. Previous studies have shown ~90% of work-related fatality cases in FARS are also found in the CFOI database [49]. Thus, the number of work-related crash fatalities may be underestimated. Thirdly, it is possible that some individuals would be incorrectly identified by crash investigation personnel as ‘not at work’ while they were ‘at work’ at time of crash; this is more likely to occur in a situation where the worker was not driving a large truck or bus, and was not wearing a uniform [11, 49]. Fourthly, this study may not capture transportation incidents which occurred outside of public roadways. Approximately 15% of occupational transportation fatalities occur on non-public roadways [4]. Occupational fatalities due to all-terrain vehicles and trucks on private property have been described in WV. Notably, several deaths have been recorded due to workers being struck by haul trucks on private mine roads [8, 9, 19]. Additionally, due to the cross-sectional nature of this study, causality cannot be determined. Lastly, because more drivers were involved in fatal work-related single vehicle collisions, the actual cause of the crash was often unknown.

## Conclusions

This study determined that work-related crashes differed greatly from non-work-related crashes in WV. These collisions also occurred in slightly different areas of the state; future studies could involve spatio-temporal modeling to discern or potentially explain why these patterns exist. Moreover, there were stark differences between in-state and out-of-state drivers killed in these collisions highlighting two distinct types of workers and subsequent crashes. Various avenues for workplace safety interventions exist, including seatbelt initiatives and drug testing policies of non-commercial drivers, which could help mitigate WV’s exorbitantly high occupational-related traffic fatality rate.
